# Long noncoding RNA HULC accelerates liver cancer by inhibiting PTEN via autophagy cooperation to miR15a

**DOI:** 10.1186/s12943-018-0843-8

**Published:** 2018-06-12

**Authors:** Xiaoru Xin, Mengying Wu, Qiuyu Meng, Chen Wang, Yanan Lu, Yuxin Yang, Xiaonan Li, Qidi Zheng, Hu Pu, Xin Gui, Tianming Li, Jiao Li, Song Jia, Dongdong Lu

**Affiliations:** 1 Research Center for Translational Medicine at Shanghai East Hospital, School of Life Science and Technology, Tongji University, Shanghai, 200092 China; 2 School of Medicine, Tongji University, Shanghai, 200092 China; 3 Tongji University School of Life Science and Technology, Shanghai, 200092 China

**Keywords:** Lnc RNA HULC, PTEN, Autophagy, miR15a

## Abstract

**Background:**

Long noncoding RNA HULC is highly up-regulation in human hepatocellular carcinoma (HCC). However, the functions of HULC in hepatocarcinogenesis remains unclear.

**Methods:**

RT-PCR, Western blotting, Chromatin immunoprecipitation (CHIP) assay, RNA Immunoprecipitation (RIP) and tumorignesis test in vitro *and* in vivo were performed.

**Results:**

HULC is negatively associated with expression of PTEN or miR15a in patients of human liver cancer. Moreover, HULC accelerates malignant progression of liver cancer cells *in vitro* and in vivo. Mechanistically, HULC increasesthe expression of P62 via decreasing mature miR15a. On the other hand, excessive HULC increases the expression of LC3 on the level of transcription and then activates LC3 through Sirt1 (a deacetylase). Notably, HULC enhanced the interplay between LC3 and ATG3. Furthermore, HULC also increases the expression of becline-1(autophagy related gene). Therefore, HULC increases the cellular autophagy by increasing LC3II dependent on Sirt1.Noteworthy, excessive HULC reduces the expression of PTEN, β-catenin and enhances the expression of SAPK/JUNK, PKM2, CDK2, NOTCH1, C-Jun in liver cancer cells. Of significance, our observations also revealed that HULC inhibited PTEN through ubiquitin–proteasome system mediated by autophagy-P62.Ultimately,HULC activates AKT-PI3K-mTOR pathway through inhibiting PTEN in human liver cancer cells.

**Conclusions:**

This study elucidates a novel mechanism that lncRNA HULC produces a vital function during hepatocarcinogenesis.

## Background

Long noncoding RNA HULC is associated with cancer progression [1, 2]. Emerging evidences suggest that HULC plays a positive function during hepatocarcinogenesis. For examples, HULC deregulates lipid metabolism in liver cancer cells [3]. Moreover, HULC promotes hepatocarcinogenesis through effecting genes of circadian rhythm [4]. In particular, HULC enhances epithelial-mesenchymal transition(EMT) to promote metastasis of liver cancer cells [5]. Furthermore, HULC inhibited apoptosis mediated by miR9 [6]. In additional, CUDR overexpression enhanced the loading of pStat3 on the promoter region of HULC which increased HULC expression in the liver cancer [7]. Of significance, CREB enhanced the HULC expression via miR372 in tumor [8]. Interestingly, IGF2BP1 knockout triggers HULC expression dependent on posttranscriptional modification [9]. In particular, HBx affect the function of HULC [10]. Moreover, some studies also showed that HULC was be able to act as a potential cancer biomarker [11–15]. Moreover, HULC silencing suppressed angiogenesis, proliferation and invasion of glioma cells dependent on anoikis [16].

Autophagy plays an important role in cellular physiological processes and tumorigenesis [17–19]. Studies showed that miR-124-3p inhibitesbreast cancercell progression by decreased cellular autophagy [20] and autophagy is increased in the skeletal muscle of cachectic cancer patients [21]. Furthermore, autophagy-related SQSTM1/p62 is involved in cellular DNA damage response [22] and autophagy descreased the abnormal chromosome number [23]. Moreover, some reports showed autophagy was associated with cellular beta 1-integrin-c-Met signaling [24], NAD + −dependent histone deacetylase Sirt1 [25], miR212-SIRT1 pathway and cellular senescence [26], Choline dehydrogenase -SQSTM1/p62 [27].

Phosphatase and tensin homolog (PTEN) belongs to a suppressor in most cancer cells [28] and inhibit tumorigenesis and cellular self-renewal [29, 30]. Studies showed PTEN was inactivated via HDAC6 inhibitor [31] and PTEN inhibited Notch signaling and PI3K-AKT-mTOR signaling axis in cancer cells [32–36] and PTEN inhibits BM1 activity [37]. However, PTEN enables the development of pre-B acute lymphoblastic leukemia [38].

In this study, our results suggested that lncRNA HULC was highly up-regulation in human hepatocellular carcinoma. Moreover, HULC accelerates malignant progression of liver cancer cells. Furthermore, HULC activates AKT-PI3K-mTOR pathway through PTEN reduction dependent on autophagy in human liver cancer cells. This study elucidates a novel tumorigenesis mechanism for HULC in liver cancer cells.

## Methods

### Patients and tissue samples

Thirty cases of liver cancer tissues used for analysis were obtained from liver cancer patients who had undergone surgery. The liver cancer tissues were fixed in formalin before embedded in paraffin. All patients were diagnosed as liver cancer according to histological examination.

### Cell lines and plasmids

Human liver cancer line Hep3B was maintained in DMEM Medium supplemented with 10% heat-inactivated fetal bovine serum (Gibco) in a humidified atmosphere of 5% CO_2_ incubator at 37 °C. Plasmid pGFP-V-RS, pCMV6-A-GFP, pCMV6-XL5-PTEN, pGFP-V-RS-PTEN, pGFP-V-RS-Sirt1, pMiR-Target were purchased from Origene (Rockville, MD 20850, USA). pEZX-MT01-PTEN-3’UTR was purchased from GeneCopeia (Rockville, MD, USA).pCMV6-AC-GFP-HULC [the HULC sequence (NR_004855) was synthesized and cloned into the cloning site ofpCMV6-AC-GFP plasmid (Origene)], pcDNA3-sirt1[the Sirt1 sequence in the Flag-SIRT1 (Plasmid #1791,Addigene) was digested and subcloned into the cloning site of pcDNA3], pcDNA3-sirt mutant [the mutant Sirt1 (H363Y) sequence in the Flag-SIRT1 H363Y (Plasmid #1792, addigene) was digested and sub-cloned into the pcDNA3] were constructed by our lab.

### Antibody, primers, probes and mimics

Anti-PCNA (Santa Cruz, Biotech), anti-Ki67 (Santa Cruz, Biotech), anti-P62 (Santa Cruz, Biotech), Anti-PTEN (Santa Cruz, Biotech), anti-pPTEN (Santa Cruz, Biotech), Anti-METTL3 (Santa Cruz, Biotech), Anti-DGCR8 (Abcam), Anti-Droha (Abcam), Anti-exportin5 (Abcam), Anti- Dicer (Santa Cruz, Biotech), Anti-Ago2 (Abcam), Anti-Notch (Santa Cruz, Biotech), Anti-JUN (Abcam), Anti-mTOR (SantaCruz, Biotech), Anti-LC3 (Santa Cruz, Biotech), Anti-RNApolII (Santa Cruz, Biotech), Anti-sirt1 (Abcam), Anti-ATG3 (Abcam), Anti-PKM2 (Santa Cruz, Biotech), Anti-CDK2 (Santa Cruz, Biotech), Anti-AKT (Abcam), Anti-mTOR (Abcam), Anti-PI3K (Abcam), Anti-survivin (Santa Cruz, Biotech), Anti -H-Ras (Santa Cruz, Biotech), Anti-BrdU (Santa Cruz, Biotech), Anti-β-actin (Abcam), anti-m6A (Santa Cruz, Biotech), anti-HistoneH3 (Abcam), anti-AcAb (Danvers, MA, USA), anti-β-catenin (Santa Cruz, Biotech), anti-pAKT (Abcam), anti-pPI3K (Abcam), anti-pmTOR (Abcam). HULCP1:5’-AACCTCCAGAACTGTGAT-3′, HULCP2:5’-CATAATTCAGGGAGAAAG-3′; PTENP1:5’-CATGACAGCCATCATCAAAG-3′; PTENP2:5’-CTGGGAATAGTTACTCCCTT-3′; LC3P1:5’AGACCGGCCTTTCAAGCAGC-3′, LC3P2:5’-CTGGGAGGCGTAGACCATAT-3′; LC3CHIPP1:5’-GCCTCCTGGGAACCAGAGAG-3′, LC3CHIPP2;5’-GCACTCTACCTTGGCGACAC-3′; H-RasP1:5’-AAGAGTGCGCTGACCATCCA-3′, H-RasP2:5’-AGAGCACACACTTGCAGCTC-3′; H-RasCHIPP1:5’-TCGGCTCCGGTCTCCAGCCA-3′, H-RasCHIPP2:5’-GCGCGGCCTACCATTGGCTG-3′; Sirt1P1:5’-GATCCTCAAGCGATGTTTG-3′, Sirt1P2:5’-ATTATTACACTATGATTTGT-3′; Pre-miR15aP1:5’-CCTTGGAGTAAAGTAGCAGC-3′, Pre-miR15aP2:5’-CCTTGTATTTTTGAGGCAGC-3′; miR15aP1:5’-TAGCAGCACATAATGGTTTGTG-3′; PrimiR15aP1:5’-AAGATCAGATCCTTGTATTT-3′, PrimiR15aP2:5’-ATTTTTTATATTCTTTAGGC-3′; β-actinP1:5’-CTTCCTTCCTGGGCATGGAG-3′, β-actinP2:5’-TGGAGGGGCCGGACTGGTCA-3′; U6P1:5’-GCTTCGGCAGCACATATACT-3′, U6P2:5’-GGAACGCTTCACGAATTTGC-3′; miR15aprobe (Northern):5’-Biotin-CACAAACCATTATGTGCTGCTA-3′; miR15aprobe (EMSA):5’-Biotin-TAGCAGCACATAATGGTTTGTG**-**3′; HULCprobe5’-Biotin-ACTCATGATGGAA-3; U6probe (Northern):5’-Biotin-ACGCTTCACGAA-3′; mimic-miR15a:TAGCAGCACATAATGGTTTGTG.

### RT-PCR

Total RNA was purified using Trizol (Invitrogen) according to manufacturer’s instructions. cDNA was prepared by SuperScript First-Strand Synthesis System (Invitrogen). PCR analysis was performed under the special conditions. β-actin was used as an internal control.

### MicroRNA detection

Total RNA was isolated from cells using Trizol (Invitrogen, Carlsbad, CA, USA) according to the manufacturer’s protocol. Real-time RT-PCR-based detection of mature miR-15a and U6 snRNA was achieved with the miRNA Detection kit (including a universe primer, U6 primers, Qiagen) and miR15a specific upsteam primers (Origene, USA).

### Western blotting

Proteins were separated on a 10% sodium dodecyl sulfate-polyacrylamide gel electrophoresis (SDS-PAGE) and transferred onto a nitrocellulose membrane. The blots were incubated with antibodyovernight at 4 °C. Following three washes, membranes were then incubated with secondary antibody overnight at 4 °C. Signals were visualized by ECL.

### RNA immunoprecipitation (RIP)

Cell lysates were incubated with A/G-plus agarose beads (Santa Cruz, Biotechnology, Inc. CA) together with the antibody or normal mouse or rabbit IgG for 4 h at 4 °C. Beads were subsequently washed and RNAs werethen isolated. RT-PCR was performed according to the manufacturer’s instructions.

### Chromatin immunoprecipitation (CHIP) assay

Cells were cross-linked with 1% (*v*/v) formaldehyde (Sigma) for 10 min at room temperature and stopped with 125 mM glycine for 10 min. Crossed-linked cells were resuspended in lysis buffer and sonicated. Chromatin extracts were immunoprecipitated with antibodies on Protein-A/G-Sepharose beads. After washing, elution and de-cross-linking, the ChIP DNA was detected by PCR.

### Cells proliferation assay

Cells growth in vitro were detected by CCK8 assay kit according to the manufacturer instruction. Cell growth curve was based on the corresponding the normalized values of OD450.

### Colony-formation efficiency assay

Cells were plated on a six well plate and the six well plate was incubated at 37 °C in humidified incubator for 10 days. Cell colonies were stained with 0.5% Crystal Violet and the colonies were counted.

### Tumorigenesis test in vivo

Four-weeks old male athymic Balb/C mice were purchased from Shi Laike Company (Shanghi, China) and maintained in the Tongji animal facilities approved by the China Association for Accreditation of Laboratory Animal Care. The athymic Balb/C mice were injected in the armpit area subcutaneously with cell suspension. The mice were observed four weeks, and then sacrificed to recover the tumors.

### Statistical analysis

Each value was presented as mean ± standard error of the mean (SEM) unless otherwise noted, with a minimum of three replicates. The results were evaluated by Student’s t-test, with *P* < 0.05 considered significant.

## Results

### HULC is negatively associated with PTEN and miR15a in human liver cancer tissues

**Fig. 1:**
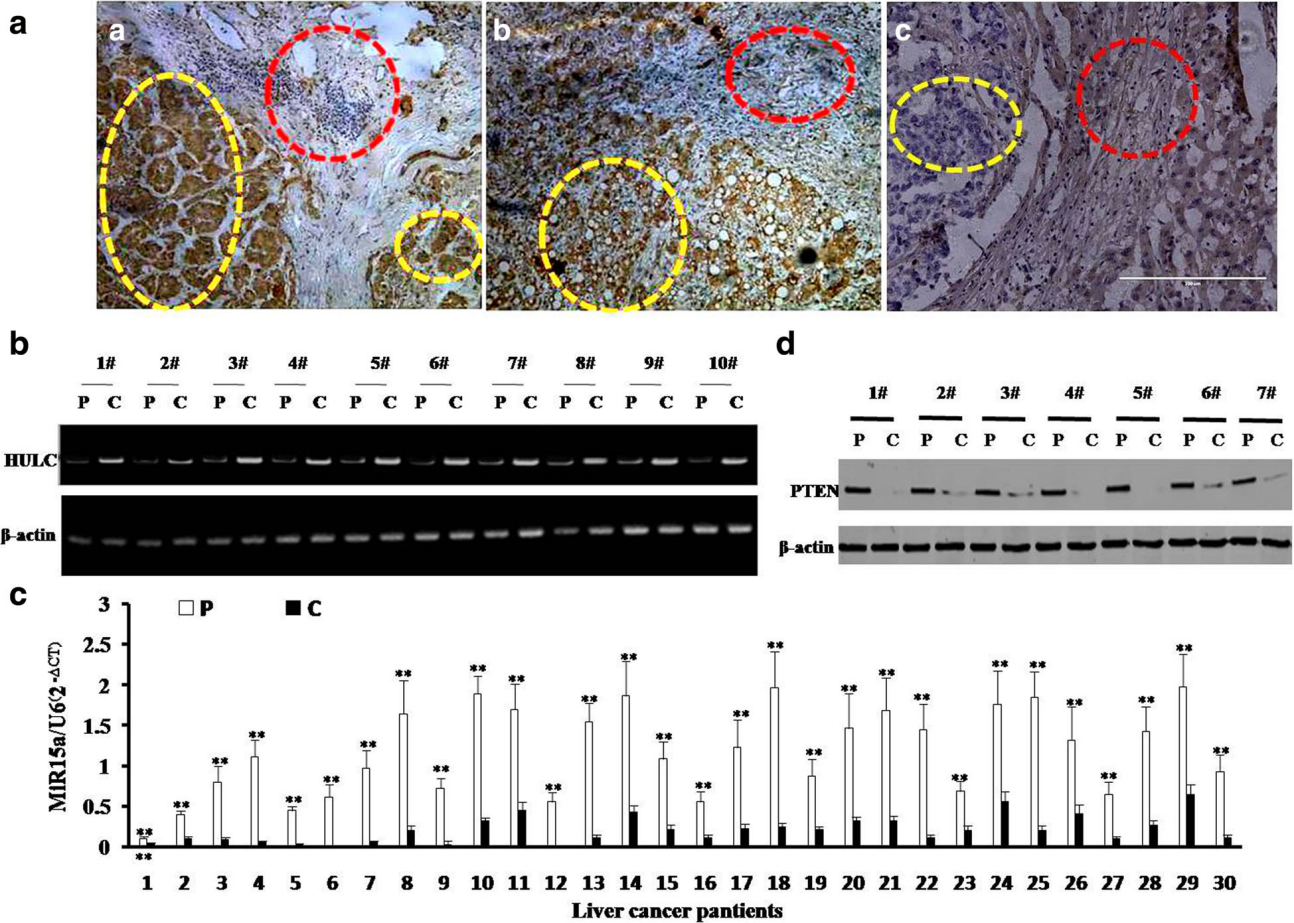
Expression analysis of HULC, miR15a, PTEN in human liver cancer tissue. **a** *(left&middle*) The representative analytic results of in situ hybridization for HULC and (*right*) immunohistochemistry staining for PTEN in human liver cancer tissue and their paired adjacent noncancerous tissues from the same patient (DAB stainning, original magnification× 100). **b** The representative analytic results of RT-PCR with HULC primers in liver cancer tissue (*c*) and its paracancerous liver tissues (*p*) respectively. β-actin as internal control. **c** The Real-time RT-PCR results of mature miR15a in liver cancer tissue (*c*) and its paracancerous liver tissues (*p*), respectively. U6 as internal control. **d** The representative analytic results of western blotting with anti-PTEN in liver cancer tissue (*c*) and its paracancerous liver tissues (*p*), respectively. β-actin as internal control

To identify the relationship between HULC and PTEN, miR15a in human liver cancer, we detected the expression of HULC, miR15a, PTEN in human hepatocarocinoma tissues. At the first time, we preformed In situ hybridization (ISH) and RT-PCR to detect HULC expression. The expression of HULC was significantly increased in liver cancer tissues compared to their adjacent noncancerous tissues (100%,*n* = 30, *P* < 0.01) [Fig. 1a (*left* and *middle*) & Fig. 1b]. Next, we detected the miR15a, the level of mature miR15a were significantly reduced in the liver cancer tissues compared to their adjacent noncancerous tissues (100%, n = 30, *P* < 0.01) (Fig. 1c). Finally, we performed immunohistochemistry staining and Western blotting to analyse PTEN expression. The expression of PTEN was significantly reduced in liver cancer tissues compared to their adjacent noncancerous tissues (100%, n = 30, *P* < 0.01) (Fig. 1a *(right*) & Fig. 1d). Significantly, there was a strong negative relevance between HULC and PTEN or miR15a. Taken Together, these observations suggest HULC was over-expressed and miR15a or PTEN was down-expressed inhuman liver cancer tissue.

### HULC accelerates growth of liver cancer cells

**Fig. 2:**
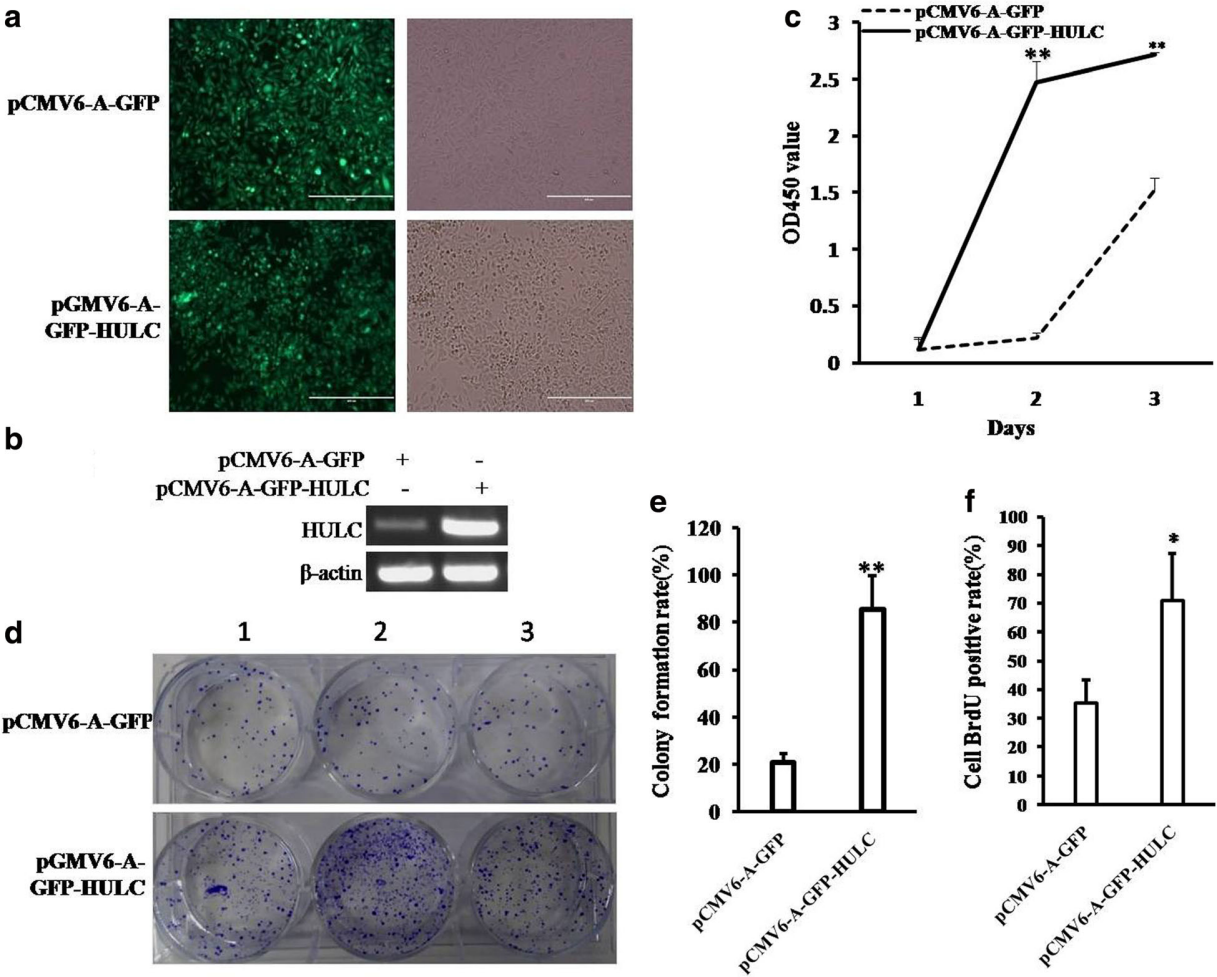
HULC promotes liver cancer cell growth in vitro. **a** The photography of the Hep3B cell lines transfected with pCMV6-A-GFP or pCMV6-A-GFP-HULC. **b** RT-PCR for HULC in HULC overexpressed control Hep3B stable cell lines; β-actin as internal control. **c** Cell proliferation assay was performed in 96-well format using the CCK8 cells proliferation kit to determine the cell viability as described by the manufacturer. Data are means of value from three independent experiments, bar±SEM. **, *P* < 0.01;*, *P* < 0.05. **d** The photography of colonies from the cell lines indicated in left. **e** Cell plate colony formation ability assay. **f** The BrdU staining assay. Data are means of value from three independent experiment, bar±SEM. **, *P* < 0.01;*, *P* < 0.05

To further investigate whether HULC promoted malignant growth of human liver cancer cell Hep3B, we first established two stable Hep3B cell lines transfected withpCMV6-A-GFP, pCMV6-A-GFP-HULC respectively. As shown in Fig. 2a & b, the expression of HULC was significantly increased in HULC overexpressing Hep3B on the transcriptional level. As shown in Fig. 2c, excessive HULC significantly increased the growth of liver cancer cell Hep3B compared to the control cells (*P* < 0.01). We further performed colony formation assay and observed a significant increase in colony formation efficiency rate in excessive HULC group compared to control (85.33 ± 14.35% vs 20.67 ± 3.83%, *P* = 0.00846 < 0.01) (Fig. 2d & e). Moreover, the BrdU positive rate is significantly increased in pCMV6-A-GFP-HULC group compared to the control cells (70.99 ± 16.44% versus 35.41 ± 8.03%, *p* = 0.0244 < 0.05) (Fig. 2f).

**Fig. 3:**
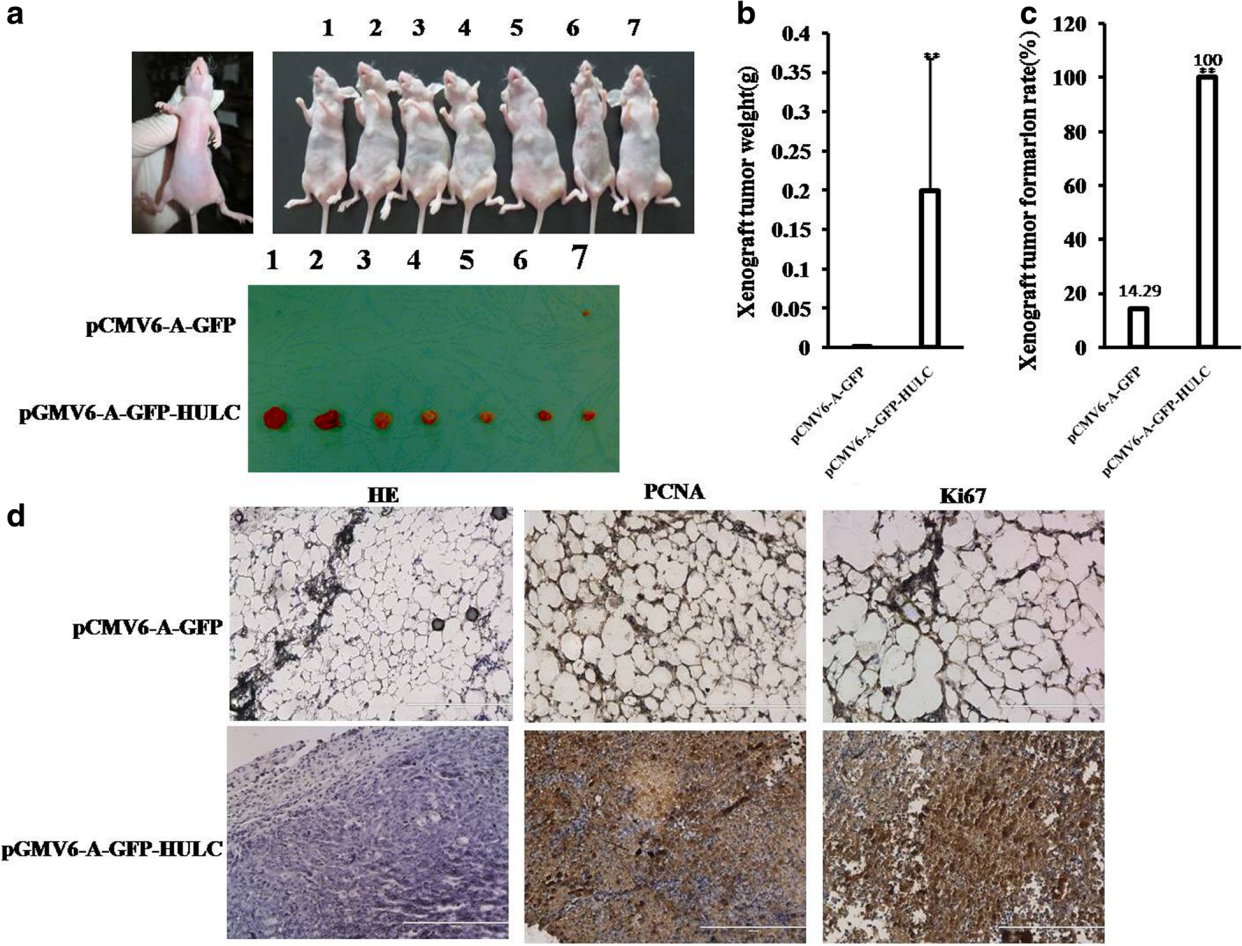
HULC promotes liver cancer cell growth in vivo*.* **a** The photography of xenograft tumors from Balb/C null mouse injected with Hep3B cells transfected with pCMV6-A-GFP,pCMV6-A-GFP-HULC subcutaneously at armpit. **b** The xenograft tumors weight (gram) in the two groups indicated in left. Data were means of value from nine Balb/c mice, mean ± SEM, *n* = 7,*, P < 0.05;**, *P* < 0.01. **c** The xenograft tumors formation rate(%) in the two groups indicated in left. **d** Hematoxylin-eosin (HE) staining of xenograft tumors (original magnification× 100). And anti-PCNA and anti-k67 immunostainningin xenograft tumor samples. (original magnification× 100)

To explore the effect of HULC on liver cancer cells in vivo, the two stable Hep3B cell lines were injected subcutaneously into Balb/C mice. As shown in Fig. 3a, b & c, when HULC was overexpressed, the average xenograft tumor weight is significantly increased compared to the corresponding control group (0.000843 ± 0.00223 g versus 0.1996 ± 0.1676 g, *P* = 0.009713 < 0.01), and when HULC was overexpressed, the xenograft tumor formation rate is significantly increased in HULC overexpressing group compared to the corresponding control group (100% versus 14.29%, *P* < 0.01). Moreover, compared to control, xenograft tumors contained more of poorly differentiated cells in pCMV6-A-GFP-HULC group compared to control group (Fig. 2d*, left*). As well as the PCNA and Ki67 were significantly higher in HULC overexpressing xenograft tumors than in the control group (Fig. 2d*, middle and right)*.

Collectively, these findings suggest that HULC accelerates malignant progression of liver cancer cells.

### HULC inhibits the expression and mature of miR15a

**Fig. 4:**
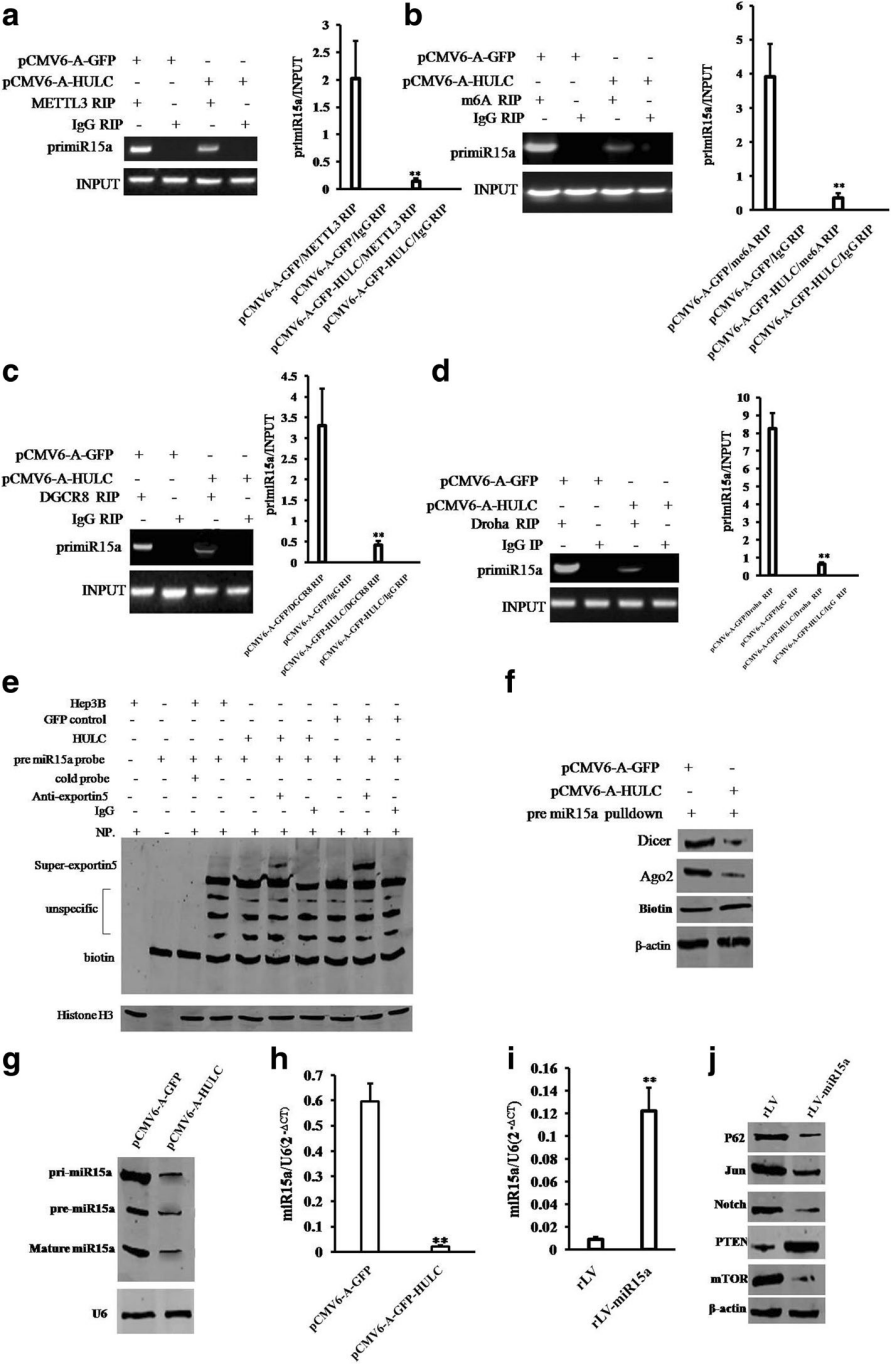
HULC inhibitsthe expression and mature miR15a. **a** (*left)* RNA Immunoprecipitation (RIP) with anti-METTL3 followed by RT-PCR with pri-miR15a primers in Hep3B cell line. IgG RIP as negative control. RT-PCR for pri-miR15a as INPUT. (*right)* quantitive RIP analysis. **b** (*left)* RIP with anti-m6A followed by RT-PCR with pri-miR15a primers in liver cancer cells. IgG RIP as negative control. RT-PCR for pri-miR15a as INPUT. (*right)* quantitative RIP analysis. **c** (*left)* RIP with anti-DGCR8 followed by RT-PCR with pri-miR15a primers in Hep3B cell line. IgG RIP as negative control. RT-PCR for pri-miR15a as INPUT. (*right)* quantitative RIP analysis. **d** (*left)* RIP with anti-Droha followed by RT-PCR with pri-miR15a primers in liver cancer cells. IgG RIP as negative control. RT-PCR for pri-miR15a as INPUT. (*right)* quantitive RIP analysis. **e** Super-EMSA (gel-shift) with biotin-pre-miR15a probe and anti-Exportin5 antibody. The intensity of the band was examined by Western blotting with anti-Bioton. HistoneH3 as internal control. **f** Biotin-pre-miR15a pulldown followed by Western blotting with anti-Dicer, anti-ago2 Biotin as INPUT and β-actin as internal control. **g** Northern blotting analysis of miR15a in liver cancer cell Hep3B cell lines. **h** The real-time PCR detection of mature miR15a in liver cancer cells. U6 as internal control. **i** The real-time PCR detection of mature miR15a in Hep3B cell lines infected with rLV and rLV-miR15a respectively. Each value was presented as mean ± standard error of the mean (SEM).**, *P* < 0.01. **j** Westernbloting with anti-P62, anti-Jun, anti-Notch, anti-PTEN, anti-mTOR. β-actin as internal control

Given that there was a strong negative relevance between HULC and miR15a expression, we wonder whether HULC influenced on the expression and mature of miR15a.Compared to the control group, HULC inhibited the binding of METTL3 (a N6-adenosine-methyltransferase) to pri-miR15a (Fig. 4a) and decreased the methylation level (me6A) of pri-miR15a (Fig. 4b). Moreover, HULC inhibited the binding of DGCR8 and Droha to pri-miR15a (Fig. 4c & d) and the binding of exportin5 and Droha to pri-miR15a (Fig. 4e). Therefore, HULC decreased the binding of Dicer and Ago2 to mature miR15a probe (Fig. 4f) and decreased the level of pre-miR15a, pri-miR15a, and mature miR15a compared to the control group (Fig. 4g & h). Furthermore, in Hep3B cell lines with excessive miR15a, (Fig. 4i), miR15a could significantly decrease the expression of P62, Notch, JUN, mTOR and increase the expression of PTEN (Fig. 4j). Together, these observations suggest that HULC inhibits expression and mature of miR15a.

### HULC promotes autophagy in liver cancer cells

**Fig. 5:**
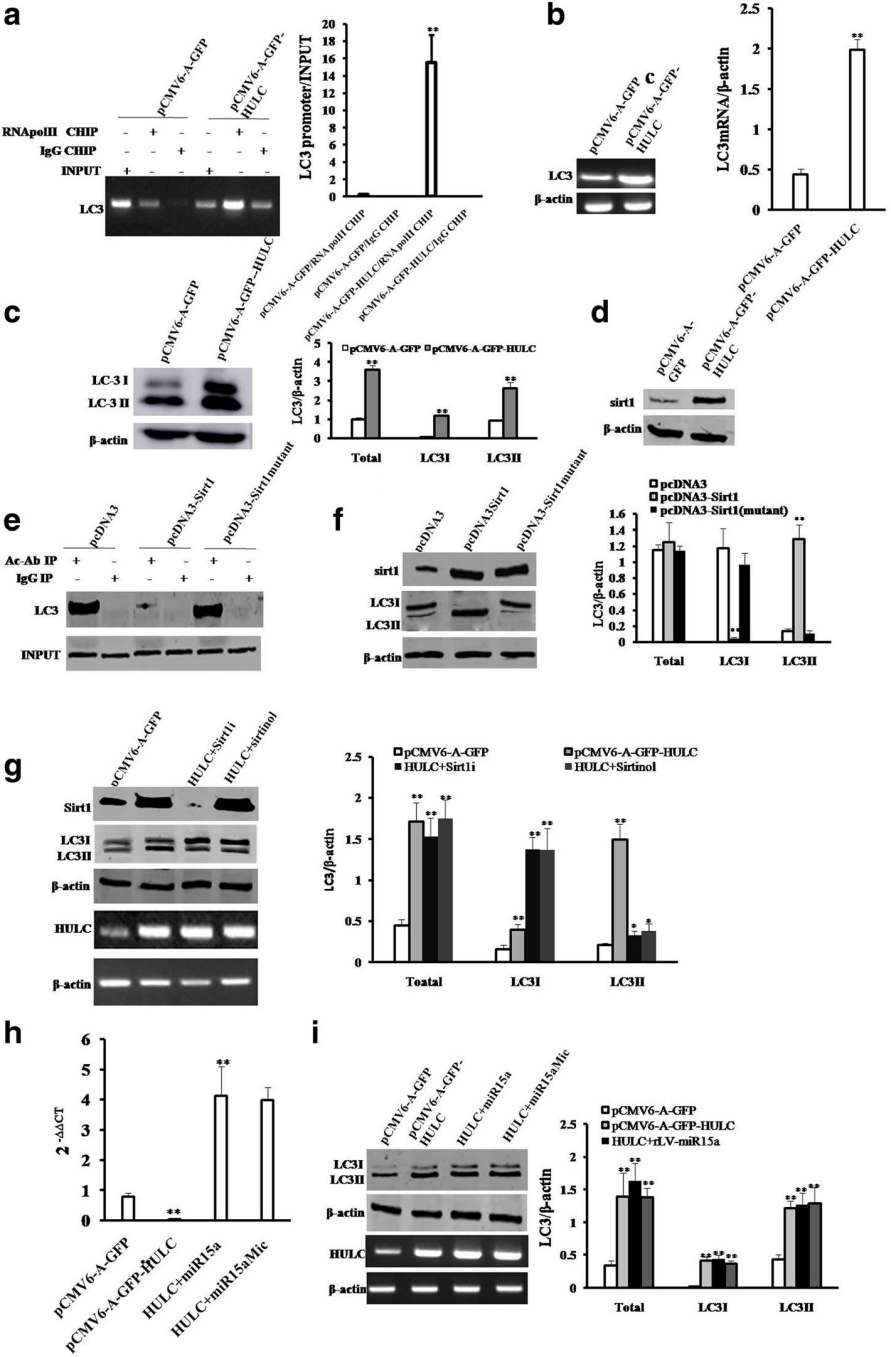
HULC promotes autophagy in liver cancer cells. **a** *(left)* Chromatin Immunoprecipitation (CHIP) with anti-RNApolII followed by PCR with LC3 promoter primers in Hep3B cell line. IgG CHIP as negative control. LG3 promoter as INPUT. (*right)* quantitative CHIP analysis. **b** *(left)* RT-PCR with LC3 primer in Hep3B cell line. (*right)* Real-time RT-PCR analysis. **c** *(left)* Western blotting with anti-LC3 in Hep3B cell line. β-actin as internal control. (*right)* density analysis of band. **d** Western blotting with anti-sirt1 in Hep3B cell line. **e** Co-Immunoprecipitation (IP) with anti-Ac Ab followed by Western blotting with anti-LC3 in Hep3B cell line. IgG IP as negative control. INPUT refers to Western blotting with. Anti-LC3. **f** *(left)* Western blotting with anti-LC3 and anti-Sirt1 in Hep3B cell lines transfected with pcDNA3,pcDNA3-Sirt1,and pcDNA3-Sirt1 mutant respectively. (*right)* density analysis of band. **g** *(left)* Western blotting with anti-LC3 and anti-Sirt1,and RT-PCR with HULC primers in Hep3B cell line. (*right)* density analysis of band. **h** Thereal-time PCR detection of mature miR15a in liver cancer cells. **i** *(left)* Western blotting with anti-LC3 and RT-PCR with HULC primers in Hep3B cell line. (*right)* density analysis of band

**Fig. 6:**
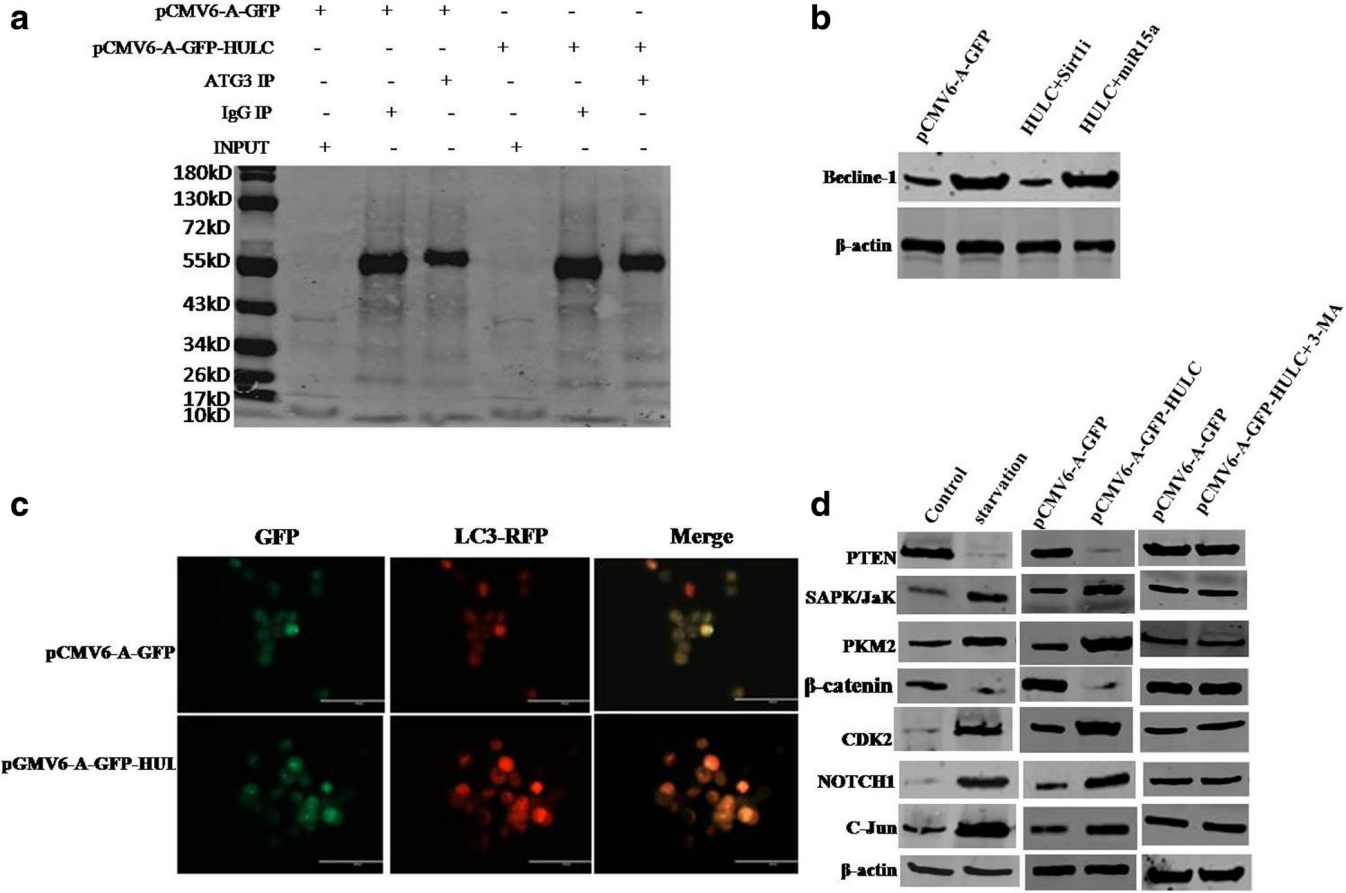
HULC alters gene expression via autophagy. **a** Co-Immunoprecipitation (IP) with anti-ATG3 followed by Western blotting with anti-LC3 in Hep3B cell line. IgG IP as negative control. INPUT refers to Western blotting with. Anti-LC3. **b** Western blotting with anti-becline-1 in Hep3B cell line. **c** Observation for autophagy (LC3-RFP) in liver cancer cells Hep3B cell line. Scale bars, 100 μm. **d** Western blotting with anti-SAPK/JaK, anti-PKM2, anti-CDK2, anti-Notch1, anti-C-Jun,anti-PTEN, anti-β-catenin in Hep3B cell lines under starvation or transfected with pCMV6-A-GFP, pCMV6-A-GFP-HULC or pCMV6-A-GFP- HULC plus 3-methyladenine (3-MA),respectively

To address whether HULC was associated with autophagy, we analyse the HULC functions on autophagy in liver cancer cells. At the first time, both the CHIP and the quantitative CHIP showed that HULC overexpression enhanced RNA polII loading on the LC3 promoter region (Fig. 5a). Furthermore, both RT-PCR and real-time RT-PCR showed that HULC overexpression increased the mRNA level of LC3 (Fig. 5b). Finally, HULC overexpression increased the expression of LC3I and LC3II (a autophagy marker) compared to control (Fig. 5c). On the other hand, HULC overexpression increased the expression of Sirt1 (Fig. 5d). Furthermore, Sirt1 could decreased the acetylation of LC3, however, the mutant Sirt1 did not result in this action (Fig. 5e). Notably, excessive Sirt1 could increase the LC3 II (a LC3 I trans-located product), however, the mutant sirt1 did not result in this action (Fig. 5f). Excessive HULC could increase the LC3I and LC3 II, however, either Sirt1 knockdown or sirt1 inhibitor fully abrogated the excessive HULC action in addition to increase LC3I (Fig. 5g). Interestingly, excessive HULC could increase the LC3I and LC3 II, however, either miR15a overexpression or miR15a mimic could not alter this action (Fig. 5h & i). Importantly, HULC enhanced the interplay between LC3 and ATG3 (Fig. 6a). Moreover, HULC increased the expression of becline-1, however, both Sirt1 knockdown could and miR15a overexpression could not abrogate this HULC action (Fig. 6b). Finally, HULC increased the autophagy of Hep3B cell (Fig. 6c). Furthermore, HULC decreased the expression of PTEN, β-catenin and increased the expression of SAPK/JUNK, PKM2, CDK2, NOTCH1, C-Jun under starvation. However, in pCMV6-A-GFP-HULC plus 3**-**methyladenine (3-MA, autophagy inhibitor) group, the expression of PTEN, β-catenin, SAPK/JUNK, PKM2, CDK2, NOTCH1, C-Jun were significantly not altered compared to control (Fig. 6d). Taken together, HULC enhances autophagy in liver cancer cells via activating Sirt1.These data prove that HULC promotes the expression of LC3 not via Sirt1, and HULC increases the LC3II dependent on Sirt1.In particular, HULC promotes autophagy by enhancing the autophagy process of the transformation from LCI to LC3 II.

### HULC inhibits PTEN through ubiquitin–proteasome system mediated by autophagy and P62

**Fig. 7:**
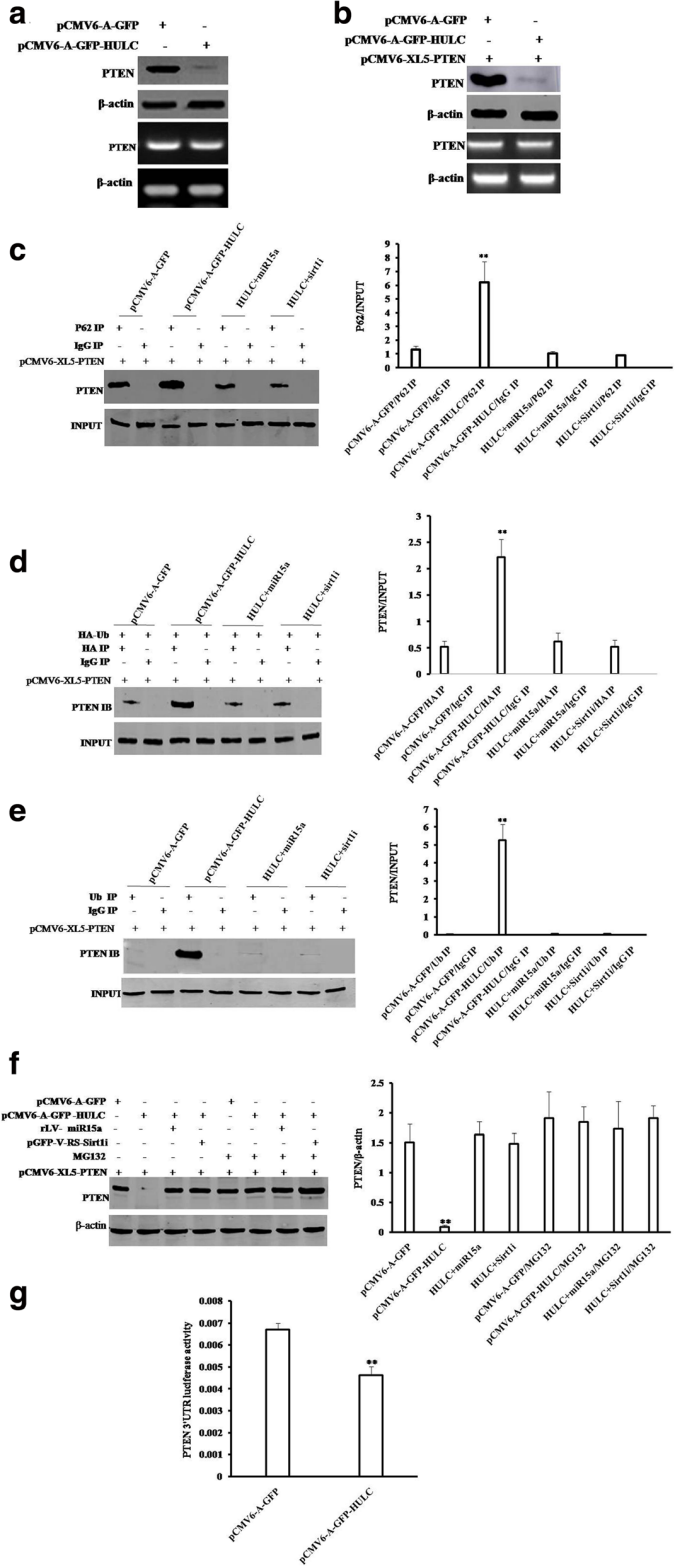
HULC inhibits PTEN through ubiquitin–proteasome system mediated by autophagy-P62. **a** Western blotting with anti-PTEN and RT-PCR with PTEN primer in Hep3B cell line. β-actin as internal control. **b** Western blotting with anti-PTEN and RT-PCR with PTEN primer in Hep3B cell lines transfected with pCMV6-A-GFP plus pCMV6-XL5-PTEN (FL), pCMV6-A-GFP-HULC plus pCMV6-XL5-PTEN (FL),respectively. **c** *(left)* Co-Immunoprecipitation (IP) with anti-62 followed by Western blotting with anti-PTEN in Hep3B cell lines transfected with pCMV6 -A-GFP plus pCMV6 -XL5-PTEN (FL), pCMV6-A-GFP-HULC plus pCMV6 -XL5-PTEN (FL), pCMV6-A-GFP-HULC plus pGFP-V-RS--Sirt1 plus pCMV6-XL5-PTEN (FL), and pCMV6-A-GFP-HULC plus pLV-miR15a plus pCMV6-XL5-PTEN (FL), respectively. (*right)* density analysis of band. **d** *(left)* Co-Immunoprecipitation (IP) with anti-HA followed by Western blotting with anti-PTEN in the Hep3B cell line. INPUT refers to Western blotting with. Anti-PTEN. (*right)* density analysis of band. **e** *(left)* Co-Immunoprecipitation (IP) with anti-HA followed by Western blotting with anti-PTEN in the Hep3B cell line. (*right)* density analysis of band. **f** *(left)* Cells were incubated with 50 μM MG132 (Sigma) for 6 h at proper after transfection. Western blotting with anti-PTEN in Hep3B cell lines transfected with pCMV6-A-GFP plus pCMV6-XL5-PTEN (FL). **g**. The assay of PTEN 3'-UTR luciferase activity in Hep3B cells infected with pCMV6-A-GFP or pCMV6-A-GFP-HULC

Given that HULC suppresses the expression of PTEN, miR15a and enhances the cell autophagy, and miR15a could inhibit the expression of 62, we wonder whether the effect of HULC on PTEN is associated with cell autophagy and miR15a. As shown in Fig. 7a, HULC inhibited the expression of PTEN on the translational level, but not on the transcriptional level. Moreover, HULC also inhibited the expression of recombinant PTEN on the translational level, but not on the transcriptional level (Fig. 7b). Furthermore, HULC enhanced the interplay between P62 and PTEN. However, both excessive miR15a and Sirt1 knockdown abrogated this HULC’s action (Fig. 7c). Surprisingly, excessive HULC promotes the ubiquitination of PTEN compared to control. However, both excessive miR15a and Sirt1 knockdown abrogated this HULC’s action (Fig. 7d & e). On the other hand, HULC inhibited the expression of PETN, and both excessive miR15a and Sirt1 knockdown abrogated this HULC’s action. In particular, HULC could not alter the expression of PTEN after the MG132 (ubiquitin– proteasome inhibitor) (Fig. 7f). In addition, we also found that HULC decrease the PTEN 3’-UTR luciferase activity (Fig. 7g). Taken together, these observations suggest that HULC inhibits PTEN through ubiquitin–proteasome system mediated by autophagy and P62.

### HULC activates AKT-PI3K-mTOR pathway by reducing PTEN in liver cancer cells

**Fig. 8:**
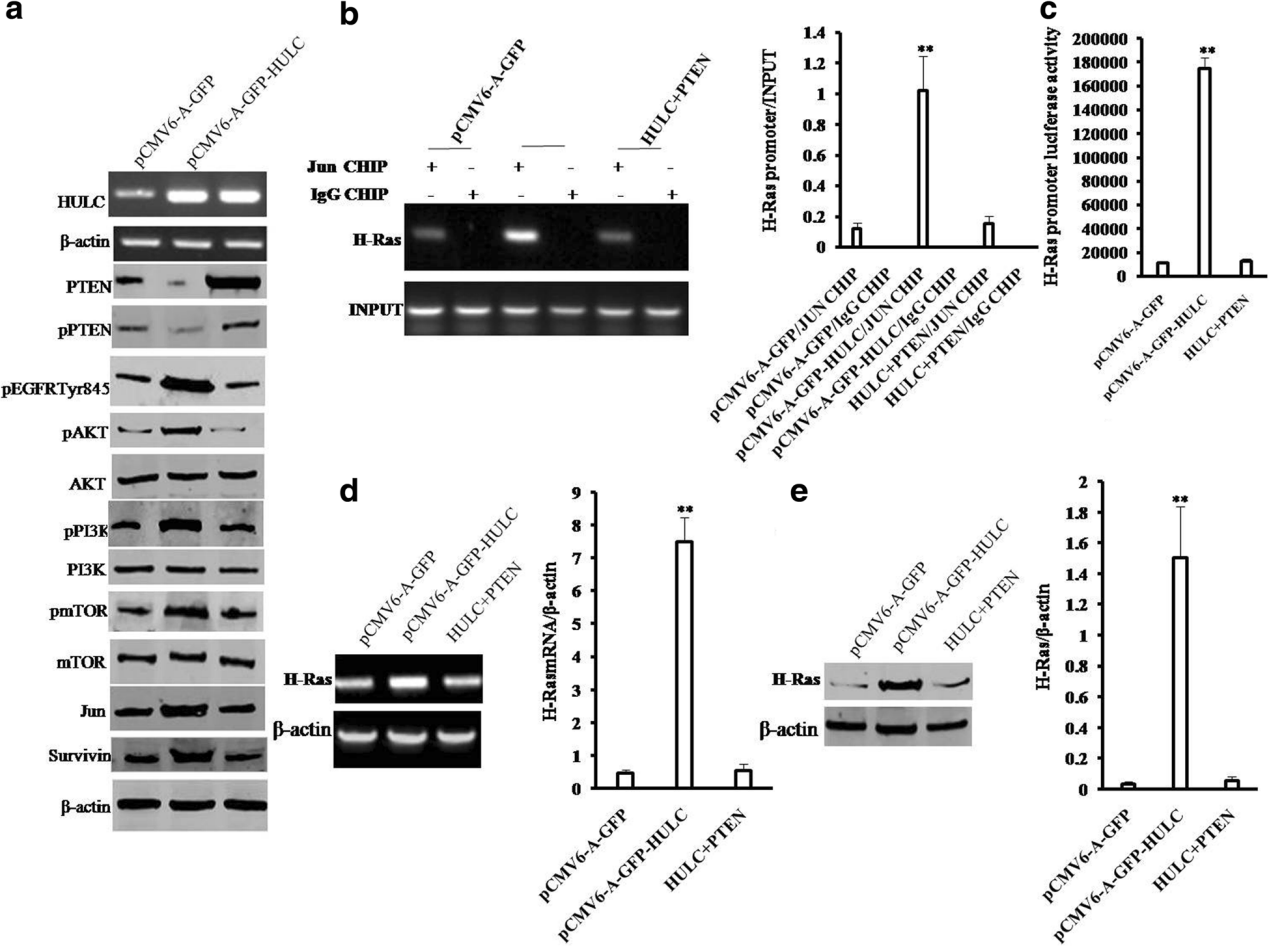
HULC activates AKT-PI3K-mTOR pathway via PTEN reduction in liver cancer cells. **a** Western blotting with anti-PTEN, anti-pPTEN, anti-EGFR (Tyr845),anti-AKT,anti-pAKT,anti-TmTOR,anti-PI3K,anti-pPI3Kanti-mTOR,anti-pmTOR, anti-JUN, anti-Survivin and RT-PCR with HULC primer in Hep3B cell lines transfected with pCMV6-A-GFP, pCMV6-A-GFP-HULC,and pCMV6-A-GFP -HULC plus pCMV6-XL5-PTEN,respectively. **b** *(left)* CHIP with anti-JUN followed by PCR with H-Ras promoter primers in Hep3B cell line. IgG CHIP as negative control. H-Ras promoter as INPUT. (*right*) quantitative CHIP. **c** The assay of H-Ras promoter luciferase activity in Hep3B. **d** *(left)* The RT-PCR with H-Ras primers in Hep3B cell line. *(right)* The real-time RT-PCR. **e** *(left)* Western blotting with anti-H-Ras in Hep3B cell line. *(right)* The density analysis of band

**Fig. 9:**
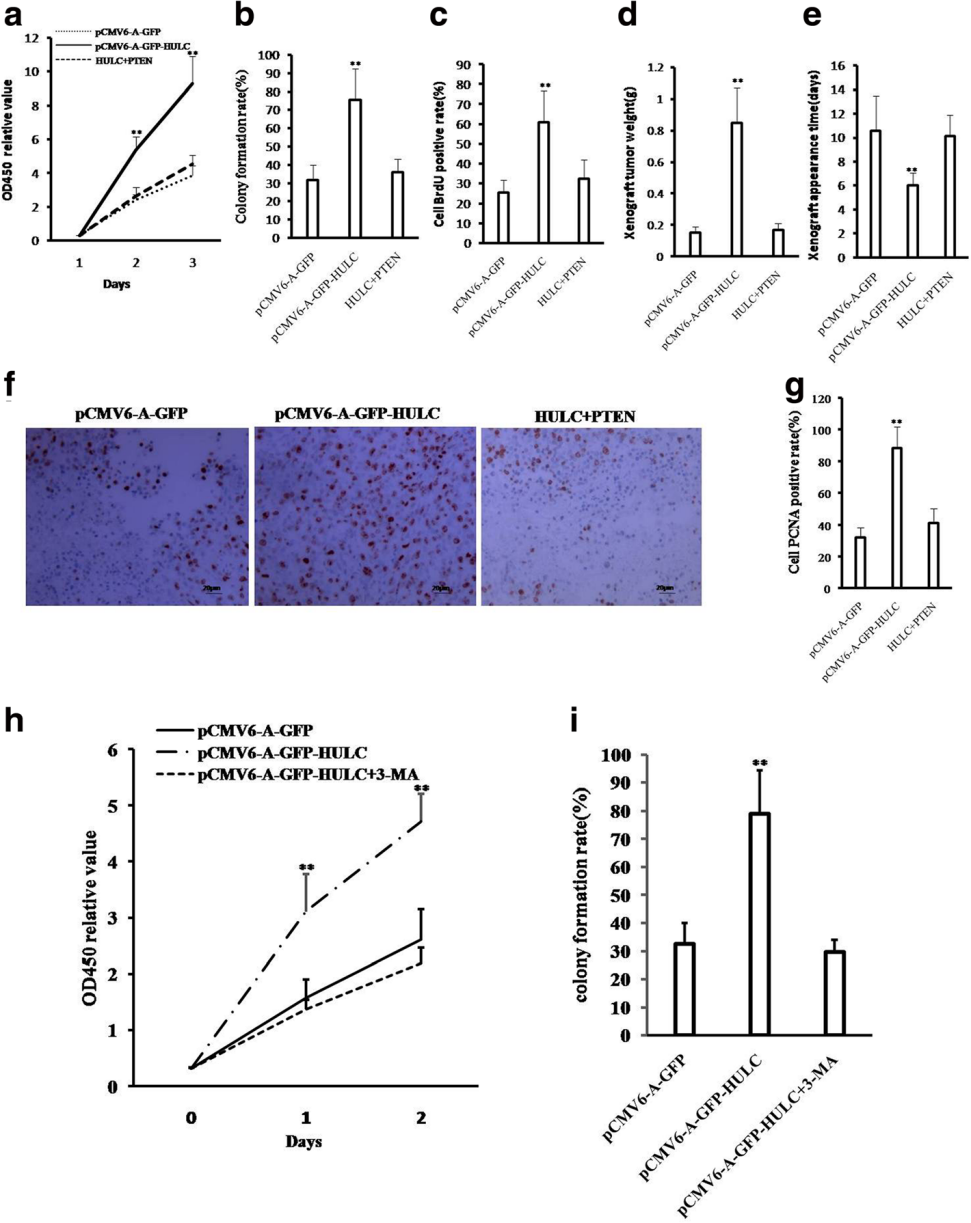
excessive PTEN abrogates the oncogenic action of HULC. **a** Cells growthassay using CCK8. **b** Cell BrdU staining assay. **c** Cells colony formation assay. **d** Tumorigenesis test in vivo. The wet weight of tumor was determined for each mouse. **e** The appearance time of tumor was determined for each mouse. **f** PCNA staining (DAB staining, original magnification× 100). **g** positive rate of PCNA staining. **h** Cells growth assay using CCK8 in pCMV6-A-GFP control group, pCMV6-A-GFP-HULC group, and pCMV6-A-GFP-HULC+ 3-Methyladenine (3-MA). **i** Cells colony formation assay in pCMV6-A-GFP control group, pCMV6-A-GFP-HULC group, and pCMV6-A-GFP–HULC + 3-Methyladenine (3-MA)

To explore whether oncogenic action of HULC is associated with PTEN, we established three stable Hep3B cell lines (pCMV6-A-GFP, pCMV6 -A-GFP-HULC, pCMV6-A-GFP-HULC plus pCMV6-XL5-PTEN). Compared to control, excessive HULC inhibits the expression and phosphorylation of PTEN. Moreover, excessive HULC increases the phosphorylation of EGFR (Tyr845), AKT, mTOR, PI3K, JUN and the expression of survivin. However, excessive PTEN abrogated the HULC’s action (Fig. 8a). Furthermore, excessive HULC increased the loading of Jun on the H-Ras promoter region. However, PTEN overexpression fully abrogated this HULC’s action (Fig. 8b). Moreover, excessive HULC increased the luciferase activity of the H-Ras promoter. However, PTEN overexpression fully abrogated the HULC’s action (Fig. 8c). Ultimately, excessive HULC increased the mRNA of H-Ras and the expression of H-Ras. However, rescued PTEN fully abrogated this HULC’s action (Fig. 8d & e). Next we detected the cell proliferation and colony formation ability. As shown in Fig. 9a, excessive HULC significantly increased the growth of liver cancer cell Hep3B compared to the control cells (*P* < 0.01). However, HULC plus PTEN did significantly not alter the growth ability of liver cancer cells (*P* > 0.05). We further performed colony formation assay and observed a significant increase in colony formation efficiency rate in excessive HULC (75.68 ± 16.78% vs 31.77 ± 8.22%, *P* = 0.00777 < 0.01). However, HULC plus PTEN did significantly not alter the colony formation rate of liver cancer cells (36.12 ± 6.85% vs 31.77 ± 8.22%, *P* = 0.2446 > 0.05) (Fig. 9b). Furthermore, HULC overexpression significantly increased the BrdU positive rate compared to the control cells (60.82 ± 15.97% versus 25.59. ± 6.04%, *p* = 0.047 < 0.05). However, HULC plus PTEN did significantly not alter the BrdU positive rate of liver cancer cells (32.37 ± 9.68% versus 25.59. ± 6.04%, *p* = 0.1966 > 0.05) (Fig. 9c). Furthermore, the three stable Hep3B cell lines were injected subcutaneously into Balb/C mice. As shown in Fig. 9d, when HULC was overexpressed, the average xenograft tumor weight increased approximately5.58 folds compared to the corresponding control group (0.848 ± 0.222 g versus 0.152 ± 0.035 g, *P* = 0.00054 < 0.01). However, HULC plus PTEN did significantly not alter the xenograft tumor weight (0.167 ± 0.044 g versus 0.152 ± 0.035 g, *P* = 0.3196 > 0.05). On the other hand, when HULC was overexpressed, the xenograft tumor appearance time was significantly decreased compared to the corresponding control group (6.017 ± 1.049 days versus 10.583 ± 2.873 days, *P* = 0.003873 < 0.01). However, HULC plus PTEN did significantly not alter the xenograft tumor appearance time (10.133 ± 1.749 days versus 10.583 ± 2.873 days, *P* = 0.403494 > 0.05) (Fig. 9e). Moreover, the PCNA was significantly higher in HULC overexpressing xenograft tumors than in the control group (88.27 ± 13.49% versus 31.83 ± 6.41%, *P* = 0.009494 < 0.01). However, HULC plus PTEN did significantly not alter the PCNA positive rate of liver cancer cells (40.97 ± 9.34%versus 31.83 ± 6.41%, *P* = 0.1397 > 0.05) (Fig. 9f & g). It suggests that HULC promotes cell growth, colony formation ability and cell growth in vivo. However, PTEN overexpression abrogated this HULC’s action. Taken together, PTEN determines oncogenic function of HULC in liver cancer cells.

### HULC promotes the growth of hepatoma cells by targeting the process of autophagy

Given that HULC promotes autophagy by enhancing the autophagy process of the transformation from LCI to LC3 II (a autophagy marker), we try to test whether that HULC promotes the growth of hepatoma cells by targeting this process. Our results showed the cell proliferation ability in vitro was significantly increased in pCMV6-A-GFP-HULC group compared to pCMV6-A-GFP control group. However, when the 3-Methyladenine (3-MA) (a autophagy inhibitor for blocking from LC3I to LC3II specifically) was added, the cell proliferation ability did not significantly alter in pCMV6-A-GFP-HULC plus 3-MA group compared to pCMV6-A-GFP control group (Fig. 9h). Moreover, the results showed the cell colony formation ability was significantly increased in pCMV6-A-GFP-HULC group compared to pCMV6-A-GFP control group (32.6 ± 7.18% vs 78.93 ± 15.33%, *P* = 0.00731 < 0.01). However, when the 3-MA was added, the cell colony formation ability did not significantly alter in pCMV6-A-GFP-HULC plus 3-MA group compared to pCMV6-A-GFP control group (32.6 ± 7.18% vs 29.55 ± 4.32%, *P* = 0.1354 > 0.05) (Fig. 9i). These observations suggest that HULC promotes the growth of hepatoma cells by targeting the process of autophagy (the transformation LCI to LCII).

## Discussion

**Fig. 10:**
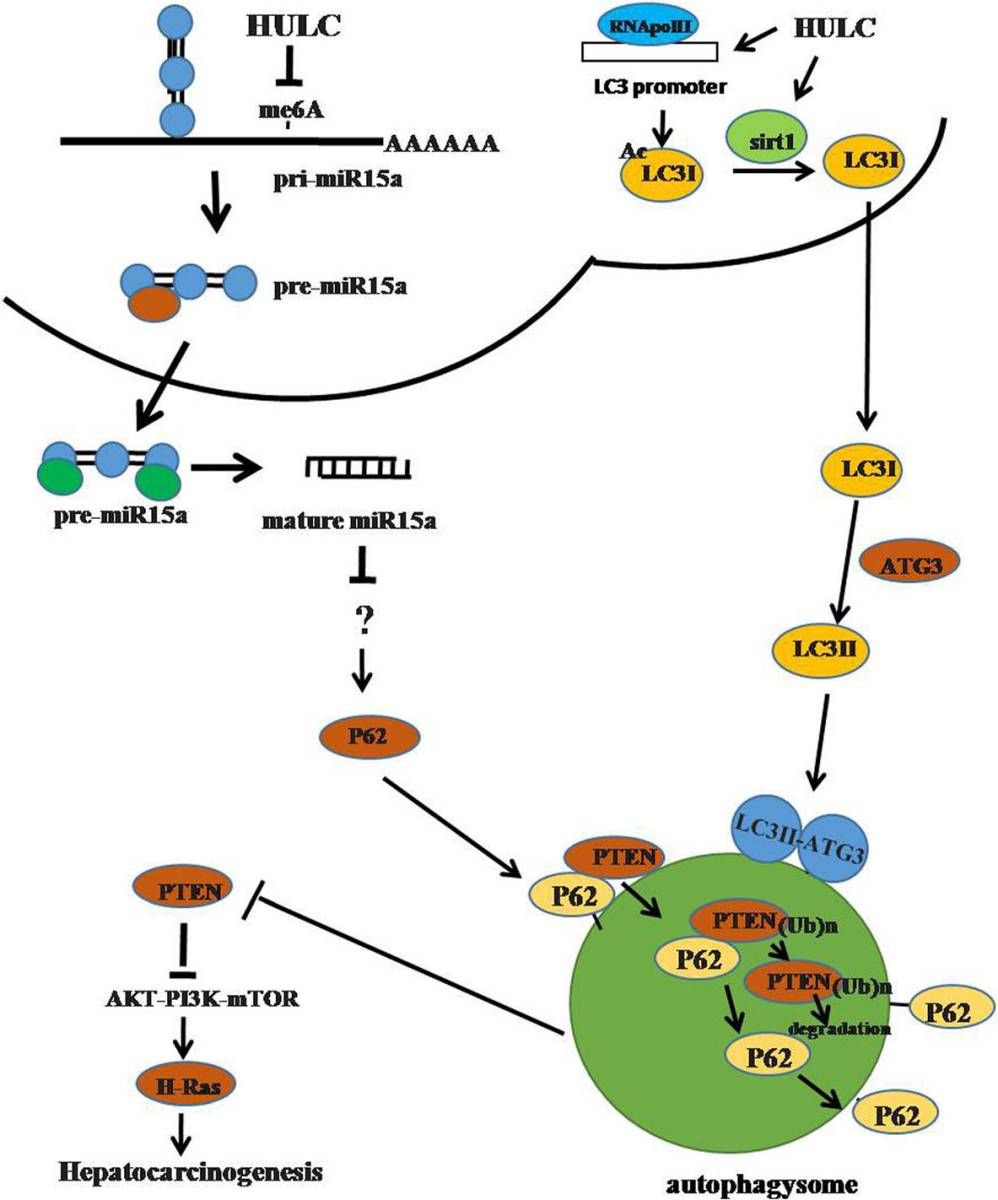
The schematic illustrates a model of Long noncoding RNA HULC promotes liver cancer cell growth by inhibition of PTEN via autophagy cooperation to miR15a. HULC decreases mature miR15a and inhibits P62 expression. Furthermore, HULC overexpression increased the expression of LC3I and LC3II (a autophagy marker) dependent on sirt1 (a deacetylase) Notably, HULC enhanced the interplay between LC3 and ATG3.Furthermore, HULC also increased the expression of becline-1(autophagy related gene). Therefore, HULC increased the cellular autophagy by activating sirt1, specifically, under starvation. Furthermore, HULC reduced the expression of PTEN, β-catenin and increased the expression of SAPK/JUNK,PKM2,CDK2, NOTCH1,C-Jun in Hep3B cells. Strikingly, HULC inhibited PTEN through ubiquitin–proteasome system mediated by autophagy-P62. Therefore, HULC activates AKT-PI3K-mTOR pathway via PTEN reduction in liver cancer cells

It has been confirmed that HULC belongs to oncogenic noncoding RNA. In this study, we indicated the effects of HULC in hepatocarcinogenesis. As shown in Fig. 10, we demonstrate that HULC is negatively associated with PTEN and miR15a expression in human liver cancer tissues. Moreover, HULC accelerates growth of liver cancer cells in vitro and *invivo*. Mechanistically, HULC decreases mature miR15a, and miR15a could indirectly decrease the expression of p62 (not a direct target of 62). Furthermore, HULC overexpression increased the expression of LC3I and LC3II (a autophagy marker) dependent on sirt1 (a deacetylase). Notably, HULC enhanced the interplay between LC3 and ATG3. Furthermore, HULC also increased the expression of becline-1(autophagy related gene). Therefore, HULC increased the cellular autophagy by activating sirt1, specifically, under starvation. Furthermore, HULC decreased the expression of PTEN, β-catenin and increased the expression of SAPK/JUNK, PKM2, CDK2, NOTCH1, C-JUN in Hep3B cells. Strikingly, our observations also revealed that HULC inhibited PTEN through ubiquitin–proteasome system mediated by autophagy-P62. Therefore, HULC activates AKT-PI3K-mTOR pathway through PTEN reduction in human liver cancer cells.

To date, accumulating evidence indicates that HULC functions in cancercinogenesis. Herein, the involvement of HULC promotion of liver cancer cell growth is supported by results from two parallel sets of experiments: 1) HULC was over-expressed in human liver cancer tissue. 2) HULC accelerates malignant growth of liver cancer cells in vitro and in vivo. Our observations demonstrated that HULC is crucial for cell growth and viability in liver cancer cells. Therefore, HULC is a strong oncogenic long noncoding RNA.

Of significance, reduction of miR15a may contribute to HULC-medicated promotion of liver cancer cell growth. Our findings in this study provide novel evidence for this action. This assertion is based on several observations: 1) HULC is negatively associated with miR15a expression in human liver cancer tissues. 2) HULC inhibited miR15 expression and its mature. 3) HULC increased the expression P62 by decreasing of mature miR15a in human liver cancer tissues. Reports suggest miR15ainhibits growth of cancer cells [39–41]. Thus, miR15a functions as a key tumor suppressor which inhibits the HULC action in liver cancer cells.

Strikingly, we demonstrated that HULC promotes autophagy in liver cancer cells and the involvement of HULC promotion of autophagy is supported by results from three parallel sets of experiments: 1) HULC increased the expression of LC3 (a autophagy marker). 2) HULC increased the expression of sirt1 andsirt1 could increased the acetylation of LC3. 3) HULC could increase the LC3I and LC3 II by activating sirt1.Notably, LC3 (microtubule-associated protein 1 light chain 3) is an essential autophagy protein and its redistribution (nuclear LC3 to the cytoplasm) is regulated through deacetylation of LC3 mediated by Sirt1 [42] and TP53INP2/DOR protein [43]. In addition, some studies found that lncRNAs and Phosphoinositides (PIs) could activates autophagy by upregulating ATG3 and ATG7 in hepatocellular carcinoma [44–46]. Accordingly, we sought to address how translocation of nuclear LC3 to the cytoplasm is regulated by HULC. In fact, we first observed that unclear LC3 is deacetylated upon excessive HULC. In particular, we also found that the translocation of nuclear LC3 to the cytoplasm is dependent on Sirt1. Therefore, we postulates that deacetylation of nuclear LC3 by Sirt1 promotes its association with ATG3 (autophagic body’s component).

Of significance, our results show that PTEN interacts with P62 and PTEN is degraded by autophagy mediated by HULC. Notably, inhibition of autophagy increases PTEN, whereas induction of autophagy decreases PTEN. In this study, we identify that autophagy-related protein p62 are required for autophagic degradation of PTEN. It suggests that autophagic degradation of PTEN through PTEN–p62 interaction is a novel tumorigenesis mechanism of HULC.

Notably, depletion of PTEN function is found inmostcancers [47, 48]. Our findings clearly showed that PTEN determines oncogenic function of HULC in liver cancer cells. This assertion is based on several observations: 1) HULC is negatively associated with PTEN expression in human liver cancer tissues. 2) HULC inhibits PTEN throughubiquitin–proteasome system mediated by autophagy-P62. 3) HULC activates AKT-PI3K-mTOR pathway via PTEN reduction in liver cancer cells.

## Conclusions

The present study depicts a novel provides evidence for HULC to play hepatocarcinogenesis roles by downregulating PTEN in liver cancer cells, which might be one of the mechanisms underlying the HULC in hepatocarcinogenesis and may have potential therapeutic significance. This understanding the novel functions of HULCin combination with autophagy will help in the development of new liver cancer therapeutic approaches.

## Abbreviations

3-MA: 3-Methyladenine
CHIP: Chromatin immunoprecipitation
HCC: Hepatocellular carcinoma
HE: Hematoxylin-eosin
LC3: microtubule-associated protein 1 light chain
lncRNA: Long noncoding RNA
METTL3: N6-adenosine-methyltransferase 3
PIs: Phosphoinositides
PTEN: Phosphatase and tensin homolog
RIP: RNA Immunoprecipitation
